# Green recovery of phenolics from bitter orange flowers: Natural deep eutectic solvent-ultrasound synergistic extraction, adsorptive purification, and UPLC/Q-TOF-MS/MS analysis

**DOI:** 10.1016/j.ultsonch.2025.107597

**Published:** 2025-10-03

**Authors:** Ziyu Lv, Jing Ma, Chi Wei, Jiaqi Wang, Dan Wang, Xinxin Cheng, Guoliang Chen, Luis A.J. Mur, Yanfeng Wang, Duo Cao

**Affiliations:** a Shaanxi Key Laboratory of Research and Utilization of Resource Plants on the Loess Plateau, College of Life Sciences, Yan’an University, Yan’an 716000 Shaanxi, China; b Department of Pathology, Xijing Hospital and School of Basic Medicine, Air Force Military Medical University, Xi’an, China; c Department of Cardiovascular Medicine, Xijing Hospital, Air Force Military Medical University, No.986 Hospital, Xi’an, China; d Department of Life Sciences, Aberystwyth University, Ceredigion SY23 3DA, UK

**Keywords:** Bitter orange flowers, Ultrasound-assisted extraction, Natural deep eutectic solvent, Adsorptive purification, Green chemistry, Sustainable extraction

## Abstract

Bitter orange flowers (BOF), a renowned medicinal and edible botanical resource, are rich in phenolic compounds with significant potential for health and industrial applications. However, efficient extraction and purification techniques for recovering bioactive phenolics remain underexplored. To address this gap, this study aimed to develop an eco-friendly integrated strategy combining natural deep eutectic solvent-ultrasound synergistic extraction (NADES-USE) with adsorptive purification for efficient recovery of total phenolics from BOF (BOF-TP). First, choline chloride-ethylene glycol (ChCl-EG) was determined to be the optimal extraction solvent from 12 synthesized NADESs and 3 common solvents. Employing Box-Behnken design (BBD), the optimized parameters for NADES-USE (38 % aqueous ChCl-EG, 18 mL/g, 345 W, 43 min, and 55 °C) resulted in a BOF-TP yield of 104.58 ± 0.34 mg/g, which was 1.30 to 2.16 times higher than those achieved with conventional solvents. Then, AB-8 resin demonstrated optimal adsorption–desorption performance for BOF-TP, with adsorption behavior strongly conforming to Langmuir isotherm and pseudo-second-order kinetic models. Thermodynamic analysis confirmed a spontaneous, exothermic physisorption process with decreasing entropy. The breakthrough curves and gradient elution curves were utilized to establish a chromatographic purification process for crude BOF-TP extracts, achieving a purity of 75.62 ± 0.95 %. Finally, a validated UPLC-Q/TOF-MS/MS method facilitated comprehensive chemical characterization and simultaneous quantification of seven bioactive compounds, serving as a basis for the quality control of purified BOF-TP extracts. In conclusion, this work demonstrates that the combination of NADES-USE and adsorptive purification offers significant potential for producing highly purified BOF-TP extract for further use in food and pharmaceutical applications.

## 1 Introduction

Bitter orange, also known as sour orange, is the fruit of *Citrus aurantium* L. (Rutaceae), originating in Southeast Asia and now widely cultivated in tropical and subtropical regions [1]. Bitter orange, along with its flowers and leaves, possesses considerable commercial value and is extensively used in the beverage, food, pharmaceutical, and cosmetics industries [2]. The bitter orange flowers, called “Dai-Dai-Hua” in Chinese, have a rich history as a food flavoring agent and in traditional Chinese medicine for treating conditions such as chest congestion, reduced appetite, vomiting, and gastric pain [3]. Recent pharmacological studies have shown that bitter orange flowers possess a range of beneficial properties, including anti-amnesic effects [4], antimicrobial [5], antioxidant [6], analgesic [7], anti-inflammatory properties [8], and the ability to alleviate dysmenorrhea [3]. Phytochemical studies indicate that bitter orange flowers are abundant in phenolics (phenolic acids and flavonoids) [8,9], alkaloids [10], essential oils [11], and polysaccharides [12]. Among these, phenolic compounds, such as gallic acid, caffeic acid, eriocitrin, naringin, hesperidin, neohesperidin, and hesperetin, have attracted considerable attention due to their significant pharmacological activity [13,14]. Consequently, to further develop bitter orange resources, it is essential to conduct more in-depth research on its flowers, with a particular emphasis on phenolics.

Phytochemical processing fundamentally requires optimized extraction protocols as a critical preliminary step for isolating target constituents from plant matrices. Traditional extraction methods, such as heat-reflux extraction and decoction, are limited by low yields, high energy consumption and costs, prolonged processing times, and the risk of thermal degradation of bioactive compounds [15,16]. Driven by the green extraction goals, numerous innovative extraction techniques have emerged in recent years to overcome the limitations of traditional methods [17]. Many studies have demonstrated that non-thermal technologies, particularly ultrasonication, offer a promising method for extracting active components. This technique utilizes high-frequency sound waves (typically between 20 and 100 kHz) to generate cavitation effects, which enhance the release of target compounds [18]. As a result, ultrasonication-assisted extraction (UAE) achieves high efficiency, low cost, reliable operation, and straightforward scalability, making it one of the most effective extraction methods available [16,19]. Ultrasound enhances the extraction process primarily through cavitation, thermal, and mechanical effects, which collectively intensify fluid flow and agitation, facilitating heat transfer and mass transfer [19]. The non-selective nature of conventional solvents (e.g., water, ethanol) facilitates co-dissolution of target analytes and matrix impurities due to their broad solvation profiles, thereby reducing extraction selectivity [20]. In addition, toxic organic extraction solvents such as methanol, ether, and acetone pose serious ecological and occupational exposure risks. In recent years, natural deep eutectic solvents (NADESs) represent a sustainable alternative to traditional solvents, aligning with green chemistry principles, and have been investigated in depth [21]. NADESs represent a class of solvents formed by mixing multiple components, typically hydrogen bond donors (HBDs) and hydrogen bond acceptors (HBAs), where cohesive hydrogen bonding drives the formation of a eutectic mixture, yielding a homogeneous liquid phase with a depressed melting point relative to the individual components [22]. With tunable physicochemical properties, facile preparation, non-toxicity, biodegradability, high recyclability, and thermal stability, NADESs demonstrate substantial potential to serve as an alternative to conventional hazardous volatile solvents in extraction applications [23]. Combining NADESs with ultrasound creates an innovative technique for the eco-friendly and sustainable recovery of phytochemicals, harnessing the benefits of both methods. NADES-USE is becoming a promising method for extracting bioactive ingredients from plant biomass, including *Paederia scandens* (Lour.) Merr. [24], *Azadirachta indica* leaves [25], *Spirulina platensis* and orange peel [26], and apple pomace [27].

Purification serves as a vital bridge connecting natural resource treasures to applications in the food and pharmaceutical industries. It is noteworthy that macroporous resins, a class of polymeric separation materials internally containing interconnected, irregularly shaped, and structurally heterogeneous pore networks with high specific surface areas, play a pivotal role in natural product purification [28]. Their surfaces are typically functionalized with nonpolar groups (e.g., styrene–divinylbenzene copolymers) or polar groups (e.g., ester, amide), enabling selective adsorption of target components through van der Waals forces, hydrogen bonding, and hydrophobic interactions [29]. Macroporous resin chromatography is distinguished among various purification methods for its environmentally friendly nature, mild operating conditions, reusability, high processing capacity, and ease of scale-up [30], making it widely applied in the purification of phenolic compounds [31]. Moreover, quality control of botanical products is crucial for verifying their safety and effectiveness due to their complex chemical compositions. UPLC/Q-TOF-MS/MS methodology provides an efficient means to identify and quantify bioactive constituents in traditional Chinese medicines, due to the great advantages of rapid analysis, high mass accuracy and high resolution. playing a crucial role in ensuring the quality control of such products [32].

Currently, there are no systematic reports on the extraction and purification of phenolics from bitter orange flowers. This study presents an integrated approach that combines NADES-UAE with macroporous resin adsorption to establish a platform for recovering total phenolics from bitter orange flowers (BOF-TP). Then, UPLC/Q-TOF-MS/MS analysis was conducted to comprehensively characterize the phenolic constituents, providing a quality assessment standard that has not been previously reported for BOF-TP extracts. The principal innovation of this work lies in the first integration of these techniques, attaining excellent extraction and purification efficiency while adhering to green chemistry principles, and is expected to facilitate the development of resources from bitter orange flowers.

## 2 Materials and methods

### 2.1 Chemicals and reagents

All compound standards were sourced from Yuanye Co., Ltd. (Shanghai, China). The Folin-Ciocalteu reagent was procured from Solarbio Co., Ltd. (Beijing, China). Chemicals for DES synthesis were obtained from Aladdin Co., Ltd. (Shanghai, China). HPD-400A, DA201, AB-8 resins were purchased from Solebo Technology Co., Ltd. (Beijing, China). HPD-100, D101, S-8, and DM301were purchased from Bon Adsorber Technology Co., Ltd. (Cangzhou, China). Their specifications are shown in Table S1. All newly acquired resins are pre-treated according to the protocols outlined in our previous study, which are detailed in the Supplementary Material [33].

### 2.2 Plant materials

Bitter orange (*Citrus aurantium* L.) flowers were collected in May 2024 from Huzhou City, Zhejiang Province, China. The samples were dried in a 50 °C oven, pulverized, screened with a 100-mesh sieve, and kept in airtight containers for future analysis.

### 2.3 Preparation of NADESs

Twelve types of NADESs, selected from the references, were synthesized by combining an HBA with an HBD at specific molar ratios, based on the method reported previously [34]. Briefly, the mixture was continuously stirred at 80 °C until a clear and uniform liquid formed, then vacuum-dried overnight to yield the NADESs. Details about the chemicals used and their molar ratios are presented in Table 1.

**Table 1:** Composition and molar ratios of HBA and HBD components in 12 DESs.

NDESs	HBA	HBD	Molar Ratio
ChCl-MA	Choline chloride	Malic acid	1:1
ChCl-OA	Choline chloride	Oxalic acid	1:1
ChCl-Lev	Choline chloride	Levulinic acid	1:2
ChCl-Gly	Choline chloride	Glycerol	1:2
ChCl-Xyl	Choline chloride	Xylitol	1:1
ChCl-EG	Choline chloride	Ethylene glycol	1:2
ChCl-TrG	Choline chloride	Triglycol	1:4
ChCl-Glu	Choline chloride	Glucose	5:2
Bet-Lev	Betaine	Levulinic acid	1:2
Bet-Gly	Betaine	Glycerol	1:1
Pro-Lev	L-Proline	Levulinic acid	1:2
Pro-Gly	L-Proline	Glycerol	2:5

### 2.4 Screening of the extraction solvent

Three common solvents (H_2_O, EtOH, 70 % EtOH) and 12 synthesized NADESs were used to extract BOF-TP. Powdered bitter orange flowers (2.0 g) were separately soaked in 50 mL of different extraction solvents. The mixtures were then subjected to ultrasonic extraction at 50 °C using a KQ-500DB ultrasonic generator (Kunshan Ultrasonic Co. Ltd., Suzhou, China) for three cycles of 30 min each. A schematic of the extraction process was shown in Fig. S1. In this study, total phenolic content was determined using the Folin-Ciocalteu assay [20], as detailed in the Supplementary Material.

### 2.5 Experimental design for optimizing the NADES-USE process

#### 2.5.1 Single-factor design

Preliminary single-factor tests were performed to assess five key parameters influencing the BOF-TP yield: water content in NEDS, liquid-to-solid ratio, ultrasonic power, extraction time, extraction temperature. Each parameter was examined individually to identify its optimal level for maximizing the BOF-TP yield, with a fixed extraction conducted twice. Single-factor experimental design is detailed in Table S2.

#### 2.5.2 Box-Behnken design

To further optimize the NADES-USE parameters significantly affecting BOF-TP yield (*Y*), response surface methodology (RSM) was employed. A Box-Behnken design (BBD) was implemented to maximize the *Y*, focusing on four critical parameters: water content (*X*_1_), liquid-to-solid ratio (*X*_2_), ultrasonic power (*X*_3_), and extraction time (*X*_4_). each at three levels (−1, 0, 1). Based on the results of the single-factor experiment, the value of each variable that produces the highest BOF-TP yield was assigned a code of 0. The actual and coded levels of the independent variables used for the BBD are presented in Table S3, along with the 29 randomized runs detailed in Table S4.

### 2.6 Static adsorption/desorption tests

#### 2.6.1 MAR screening

Batch adsorption studies were performed in 200 mL conical flasks, each containing 40 mL of BOF-TP solution (2.0 mg/mL) and 1.0 g (±0.01 g) of tested resins (D101, HPD-100, AB-8, HPD-400A, S-8, DM301, and DA201). The suspension was subjected to isothermal adsorption (25 °C) for 12 h under continuous stirring (120 rpm). The adsorbed phenolic compounds were desorbed using H_2_O, followed by 40 mL of 95 % EtOH at 120 rpm and 25 °C. The ideal resin was identified through a comprehensive evaluation of adsorption capacity (*Q_e_*, mg/g), desorption capacity (*Q_d_*, mg/g), and desorption rate (*D*), quantified using the following expressions:

(1) $$Q_{e}=\frac{\left(C_{0}-C_{e}\right)V_{s}}{m}$$

(2) $$Q_{d}=\frac{C_{d}V_{d}}{m}$$

(3) $$D=\frac{C_{d}V_{d}}{\left(C_{0}-C_{\text{e}}\right)V_{s}}\times 100\%$$

where *C*_0_ and *C_e_* (mg/mL) indicate the initial and equilibrium concentrations of BOF-TP respectively; *C_d_* (mg/mL) refers to the concentration of BOF-TP in the desorbed solution; *V_s_* and *V_d_* (mL) represent the volumes of the sample and desorption solutions, respectively; and *m* is set at 1 g.

#### 2.6.2 Adsorption isotherm

The adsorption isotherm studies were conducted using 40 mL of extract solution with BOF-TP concentrations ranging from 0.5 to 3.0 mg/mL, along with 1 g of AB-8 resin in 200 mL conical flasks. Following 5 h of orbital shaking at 120 rpm and temperatures between 25 and 55 °C, the equilibrium data were analyzed using the Langmuir (Eq. (4)), Freundlich (Eq. (5)), and Temkin (Eq. (6)) isotherm models.

(4) $$\frac{C_{e}}{Q_{e}}=\frac{1}{K_{L}Q_{m}}+\frac{C_{e}}{Q_{m}}$$

(5) $$\ln Q_{e}=\frac{1}{n}\ln C_{e}+\ln K_{F}$$

(6) $$Q_{e}=B_{T}\ln K_{T}+B_{T}\ln C_{e}$$

where *Q_m_* (mg/g) denotes the maximum adsorption capacity; *K_L_* (L/mg), *K_F_* [(mg/g)(L/mg)^1/^*^n^*], and *K_T_* (L/mg) represent the constants for the Langmuir, Freundlich, and Temkin models, respectively; and *B_T_* (J/mol) denotes a constant corresponding to the heat of adsorption*.*

#### 2.6.3 Adsorption thermodynamic parameters

Gibbs free energy change (Δ*G*), enthalpy change (Δ*H*), and entropy change (Δ*S*) for BOF-TP adsorption onto AB-8 resin were determined based on isotherm analysis data, with their quantitative relationships expressed by Eqs. (7), (8).

(7) $$\Delta G=-RT\ln K_{\mathrm{eq}}$$

(8) $$\ln K_{\mathrm{eq}}=-\frac{\Delta H}{\mathrm{RT}}+\frac{\Delta S}{R}$$

where *K_eq_* denotes the equilibrium constant, *R* (8.314 J/mol K) refers to the universal gas constant, and *T* (K) represents the absolute temperature.

#### 2.6.4 Adsorption kinetics

Batch adsorption experiments were conducted by combining 40 mL of extract solution (2.0 mg/mL of BOF-TP) with 1 g of AB-8 resin in a 200 mL conical flask. The mixture was agitated at 100 rpm at 25 °C for 4 h, and the adsorption capacity (*Q*_t_, mg/g) was measured at predetermined time intervals. Subsequently, the kinetic behavior of BOF-TP adsorption onto ADS-17 resin was evaluated using pseudo-first order (PFO, Eq. (9), pseudo-second order (PSO, Eq. (10)), and intraparticle diffusion (IPD, Eq. (11)), with their mathematical representations as follows:

(9) $$\ln\left(Q_{e}-Q_{t}\right)=-k_{1}t+\ln Q_{e}$$

(10) $$\frac{1}{Q_{t}}=\frac{1}{k_{2}Q_{e}^{2}t}+\frac{1}{Q_{e}}$$

(11) $$Q_{t}=k_{i}t^{\text{1/2}}+C_{i}$$

where *k*_1_ (min^−1^), *k*_2_ [g/(mg min)], and *k*_i_ [mg/(g min^1/2^)] represent the rate constants for the PFO, PSO, and IPD models, respectively. *C_i_* (mg/g) reflects the influence of boundary layer thickness.

### 2.7 AB-8 resin column chromatography

The AB-8 resin was placed into a glass column with a diameter of 20 mm, resulting in a diameter-to-height ratio of 1:6.5 and an approximate packed bed volume (BV) of 40 mL. For adsorption studies, crude extracts with 2.0 mg/mL BOF-TP were fed into the column at 1, 2, 3, and 4 BV/h. During sample loading, the effluent was continuously collected and monitored, with the breakthrough point identified When the BOF-TP concentration in the effluent achieved 10 % of the initial sample concentration [35]. After reaching the breakthrough point, the column was eluted sequentially using H_2_O and 10 % EtOH, 40 % EtOH, 70 % EtOH, and 95 % EtOH, respectively, with each step containing 5 BV at 2 BV/h.

### 2.8 UPLC/Q-TOF-MS/MS analysis

#### 2.8.1 Analysis conditions

The chromatographic separation was carried out on an Agilent 1290 Infinity II ultra-high performance liquid chromatography system utilizing a ZORBAX Eclipse Plus C18 analytical column (2.1 × 100 mm, 1.8 μm) under ambient temperature conditions. A binary solvent system was employed for the analysis, comprising 0.1 % formic acid (mobile phase A) and 0.1 % formic acid in acetonitrile (mobile phase B), delivered at a constant flow rate of 0.3 mL/min. The gradient elution profile was programmed as follows: initial 20 % B (0–5 min), linear increase to 30 % B (5–6 min), followed by gradient elevation to 75 % B (6–8 min), subsequent return to initial conditions (8), (9) min), and final column equilibration (9–9.5 min). Sample introduction was performed with a 0.5 μL.

The above LC system was interfaced with an Agilent G6546 Q-TOF spectrometer using an ESI source for MS detection. Mass data acquisition was performed in auto MS/MS mode with a scan rate of 2 spectra per second. Nitrogen served as the drying gas, delivered at a nebulizer pressure of 35 psi with a flow rate of 8 L/min (320 °C). The sheath gas was maintained at 350 °C with a flow rate of 11 L/min. Capillary and nozzle voltages were set to 3,500 V and 1,000 V, respectively. Collision energies of 15 eV and 35 eV were employed for MS/MS fragmentation. All measurements in positive ionization mode were conducted using a *m*/*z* of 50–800 for both MS and MS/MS scans.

#### 2.8.2 Method validation

Based on our previous report [36], the method validation primarily focused on linearity, linear response range, limit of detection (LOD), limit of quantification (LOQ), intra- and inter-day precisions, and recovery.

### 2.9 Statistical analysis

All experiments were performed in triplicate, and the results were expressed as the mean ± standard deviation. RSM design and corresponding analyses were conducted using Design-Expert 13 (Stat-Ease, Inc., Minneapolis, MN, USA). Adsorption data were processed and visualized using Origin 2024 (OriginLab Corp., Northampton, MA, USA).

## 3 Results and discussion

### 3.1 Selection of the best extraction solvent

**Fig. 1:**
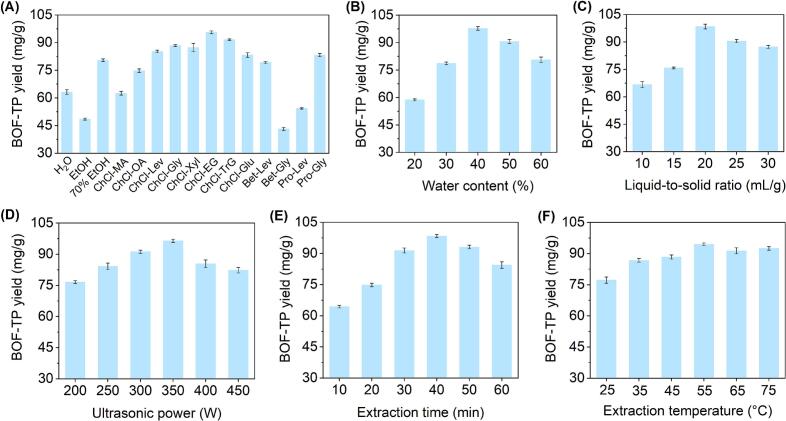
Effect of different extraction process parameters on BOF-TP yield: Extraction solvents (A), water content (B), liquid-to-solid ratio (C), ultrasonic power (D), extraction time (E), and extraction temperature (F).

In this study, choline chloride, betaine, and L-proline served as HBAs, whereas sugars, acids, and alcohols were selected as HBDs for the preparation of DESs. Fig. 1A presents the yield of BOF-TP extracted using various DESs containing 50 % (*v*/*v*) water, alongside a comparison with three commonly employed solvents. Among the three conventional solvents, 70 % EtOH exhibited the highest extraction efficacy, whereas pure EtOH produced the lowest BOF-TP yield. Among the twelve synthesized solvents, ChCl-EG exhibited the highest BOF-TP yield at 95.63 ± 0.74 mg/g, followed by ChCl-TrG at 91.61 ± 0.35 mg/g and ChCl-Gly at 88.36 ± 0.52 mg/g. These results indicated that ChCl-based DESs were more effective in extracting phenolic compounds from BOF, aligning with previous studies [37,38]. The structure of DESs plays a crucial role in the extraction efficiency of target compounds, as it directly influences inherent properties such as polarity, physicochemical interactions and solubility [37]. In accordance with the “like dissolves like” principle, the polarity of DESs is crucial for extraction efficiency. Among the tested solvents, ChCl-EG initially demonstrated favorable properties, highlighting its suitability as a green solvent for further research.

### 3.2 Single factor experimental analysis

As shown in Fig. 1B, the BOF-TP yield increased with water content from 20 % to 40 % in the ChCl-EG solution, but a further increase led to a marked decline. Similar findings have also been observed in the extraction of phenolics from foxtail millet bran [39] and apple pomace [40] using NADES-USE. An appropriate water content should simultaneously reduce the viscosity of the solvent and enhance mass transfer, thereby increasing the extraction yield of the target compounds [39]. As illustrated in Fig. 1C, raising the liquid-to-solid ratio from 10 to 20 mL/g improved the BOF-TP yield, while further increases negatively impacted the yield. Comparable results were noted in the extraction of phenolics from broccoli leaves [41] and *Citrus reticulata* Blanco peels [42]. In general, an insufficient volume of solvent may lead to incomplete extraction, whereas an excessive volume not only complicates the procedure and increases waste, but also attenuates ultrasonic energy [20]. As illustrated in Fig. 1D, increasing the ultrasonic power within the range of 200–350 W elevated the BOF-TP yield, whereas further increases in power had a negative impact on the yield. A similar result was observed during the extraction of phenolics from broccoli leaves [41]. This was due to a moderate increase in ultrasonic power, which improved extraction yield through enhanced solvent-sample interaction. However, excessive power degraded target compounds, reducing yield and quality [20]. As presented in Fig. 1E, increasing the extraction time to the 10–40 min range significantly improved the BOF-TP yield, whereas further prolongation resulted in a decrease. Similar findings have also been reported in the NADES-USE of phenolics from foxtail millet bran [39] and broccoli leaves [41]. While a moderate extension of extraction time generally favored higher yields, excessive durations promoted structural degradation of phenolics and increased the co-extraction of impurities [31]. As shown in Fig. 1F, increasing the extraction temperature from 25 to 55 °C resulted in a slight rise in BOF-TP yield, which then decreased marginally at higher temperatures. On the whole, temperature played a less significant role in affecting BOF-TP yield. Therefore, 55 °C was selected as the optimal extraction temperature.

### 3.3 RSM-based optimization of the NADES-USE procedure

#### 3.3.1 Model fitting

Following the single-factor tests, four key parameters, water content (*X*_1_), liquid-to-solid ratio (*X*_2_), ultrasonic power (*X*_3_), and extraction time (*X*_4_), were chosen for optimization via RSM, aiming to maximize the yield of BOF-TP (*Y*). A quadratic polynomial model was derived through multiple regression of the BBD results and was presented in coded factors in the Supplementary Material. ANOVA results (Table 2) confirmed the quadratic model was highly significant (*F* = 84.06, *p* < 0.0001). This indicated that the probability of obtaining such a high *F*-value by chance was only 0.01 % due to noise. An *F*-value of 3.27 was obtained for the “Lack of Fit”, indicating that it was not significant compared to the pure error (*p* = 0.1327), which was desirable as it demonstrated that the established models effectively revealed the associations between the response and the independent variables [43]. Additionally, the linear terms for *X*_1_, *X*_2_, *X*_3_, and *X*_4_, along with the interaction terms *X*_1_*X*_3_, *X*_2_*X*_3_, and *X*_3_*X*_4_, were statistically significant (*p* < 0.05), suggesting that these variables and their respective interactions had a notable impact on BOF-TP yield. The predicted *R*^2^ value of 0.9377 closely matched the adjusted *R*^2^ of 0.9765, indicating strong consistency between the model and the experimental results across the examined variable range [44].

**Table 2:** ANOVA for quadratic model.

Source	Sum of Squares	df	Mean Square	*F*-value	*P*-value
Model	1262.20	14	90.16	84.06	<0.0001
*X*_1_	163.84	1	163.84	152.76	<0.0001
*X*_2_	191.28	1	191.28	178.34	<0.0001
*X*_3_	7.08	1	7.08	6.60	0.0222
*X*_4_	53.30	1	53.30	49.69	<0.0001
*X*_1_*X*_2_	3.59	1	3.59	3.35	0.0887
*X*_1_*X*_3_	16.24	1	16.24	15.14	0.0016
*X*_1_*X*_4_	4.56	1	4.56	4.25	0.0583
*X*_2_*X*_3_	28.36	1	28.36	26.44	0.0001
*X*_2_*X*_4_	1.38	1	1.38	1.29	0.2756
*X*_3_*X*_4_	5.04	1	5.04	4.70	0.0479
*X*_1_^2^	587.18	1	587.18	547.47	<0.0001
*X*_2_^2^	147.94	1	147.94	137.93	<0.0001
*X*_3_^2^	283.14	1	283.14	263.99	<0.0001
*X*_4_^2^	110.54	1	110.54	103.07	<0.0001
Residual	15.02	14	1.07		
Lack of Fit	13.38	10	1.34	3.27	0.1327
Pure Error	1.64	4	0.4096		
Cor total	1277.21	28			

*Notes*: *X*_1_ – Water content; *X*_2_ – liquid-to-solid ratio; *X*_3_ – ultrasonic power; *X*_4_ – extraction time; df – degrees of freedom.

#### 3.3.2 Response surface analysis

**Fig. 2:**
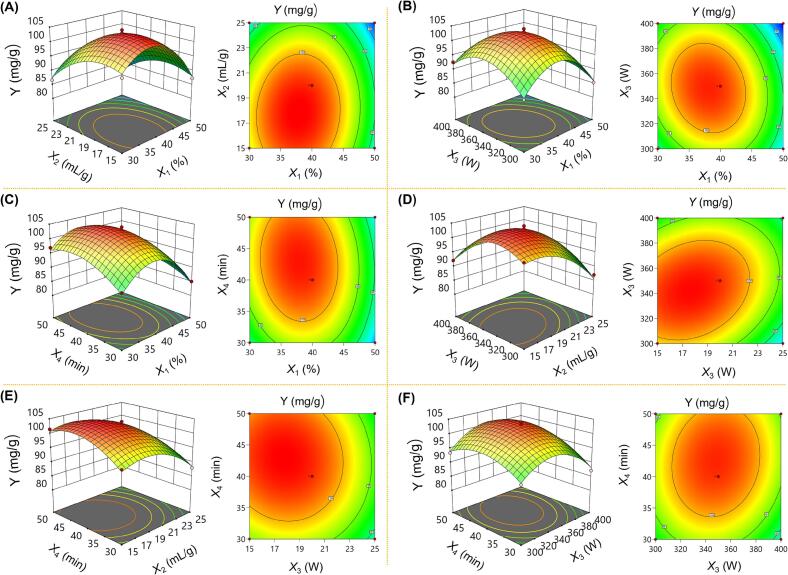
Response surface and contour plots depicting pairwise interactions between independent variables affecting BOF-TP yield.

As shown in Fig. 2, variables *X*_1_, *X*_2_, *X*_3_, and *X*_4_ had a pronounced effect on the BOF-TP yield, which was in accordance with the results obtained from ANOVA. The BOF-TP yield consistently increased to a certain point and then declined as the values of these variables rose, a trend that was consistent with the observations from the single-factor experiments. It was evident that the interaction between *X*_2_ and *X*_3_ had the most significant effect on BOF-TP yield, followed by the interactions between *X*_1_ and *X*_3_, and between *X*_3_ and *X*_4_. RSM analysis indicated that the optimal combination of independent variables for maximizing BOF-TP yield included a water content of 37.75 %, a liquid-to-solid ratio of 17.57 mL/g, ultrasonic power of 345.19 W, and an extraction time of 43.06 min.

#### 3.3.3 Model verification

To validate the applicability of the developed prediction model under the control precision requirements of actual production, validation experiments were conducted using the following modified parameters: 38 % water in ChCl-EG solution, liquid-to-solid ratio of 18 mL/g, ultrasonic power at 345 W, extraction duration of 43 min, and extraction temperature of 55 °C. Triplicate validation tests yielded a BOF-TP content of 104.58 ± 0.34 mg/g, demonstrating strong agreement with the predicted value of 104.70 mg/g. These results robustly support the practical utility of the prediction model established in this study.

### 3.4 Resin screening

**Fig. 3:**
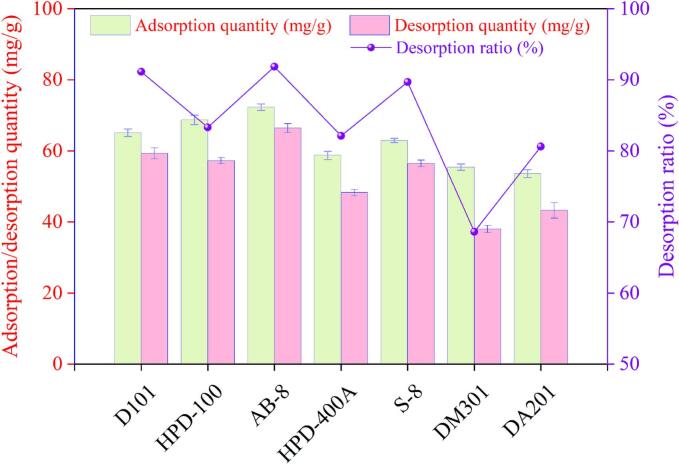
Adsorption/desorption capacities and desorption ratios of seven resins for BOF-TP.

As presented in Fig. 3, the AB-8 resin possessed the greatest adsorption capacity for BOF-TP at 72.30 ± 0.91 mg/g, followed by HPD-100 at 68.73 ± 1.33 mg/g, D101 at 65.09 ± 1.07 mg/g, and lower capacities for DA-201 and DM301 at 53.64 ± 1.14 mg/g and 55.42 ± 0.87 mg/g, respectively.The findings revealed that weak-polar and non-polar resins exhibited significantly higher adsorption capacities for BOF-TP than their strong-polar, polar, and medium-polar counterparts. This result underscored the considerable influence of resin polarity on adsorption performance, which was consistent with the principle of “like dissolves like” in chemistry [45]. Furthermore, a comparison of the non-polar resins D101 and HPD-100, as well as the medium-polar resins HPD-400A and S-8, revealed that, among resins with comparable polarity, those with greater surface area demonstrated enhanced adsorption performance. Comparable results were also observed in the purification of phenolics from *Ascophyllum nodosum* using macroporous resins [46]. S-8, D101, and AB-8 resins, possessing larger pore diameters, achieved desorption ratios of 89.70 %, 91.14 %, and 91.85 %, respectively, highlighting the role of larger pore diameters in improving desorption efficiency. It was noteworthy that AB-8 resin exhibited a high desorption capacity despite having smaller pore diameters than S-8 and D101 resins. This was likely attributable to the larger specific surface area and stronger binding affinity of S-8 and D101 resins for the target phenolic compounds, both of which can significantly hinder the desorption process [47]. Thus, AB-8 resin emerged as the best option for enriching BOF-TP due to its superior performance in adsorption and desorption.

### 3.5 Adsorption isotherms

**Fig. 4:**
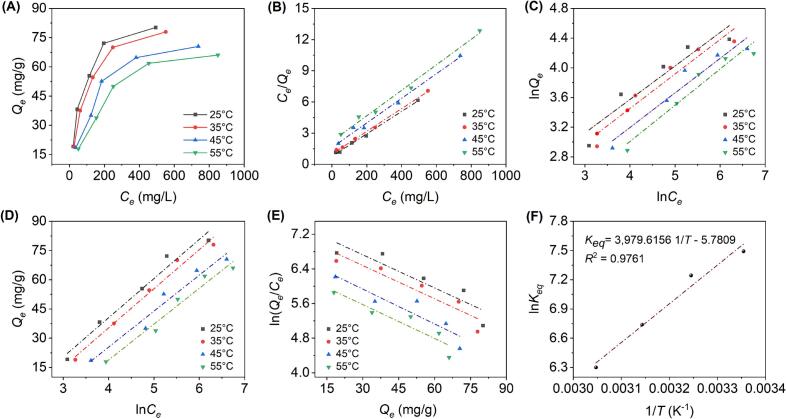
Adsorption isotherms of BOF-TP on AB-8 resin (A) at 25, 35, 45, and 55℃; Linear fitting based on the Langmuir model (B), Freundlich model (C), and Temkin model (D); Plots of ln(*Q_e_*/*C_e_*) versus *Q_e_* (E) and the plot of ln*K_eq_* versus 1/*T* (F).

As shown in Fig. 4A, the *Q_e_* of the AB-8 resin for BOF-TP decreased markedly as the temperature increased from 25 to 55 °C, indicating that the adsorption process was exothermic. Moreover, *Q_e_* increased progressively with increasing initial concentrations of BOF-TP (*C*_0_), which was likely due to the greater availability of phenolic molecules to occupy the binding sites on the resin [45]. However, when the initial concentration (*C*_0_) was increased to 2.0 mg/mL, a distinct inflection point was observed in *Q_e_*, with the rate of increase declining markedly. This phenomenon might be attributed to the gradual saturation of available adsorption sites on the resin, resulting in a diminished capacity for further adsorption at higher concentrations [33]. Therefore, a crude extract solution with a *C*_0_ of 2.0 mg/mL was used for subsequent study.

Isotherm data were fitted to the Langmuir, Freundlich, and Temkin models, with the corresponding fitting curves shown in Fig. 4B–D. As presented in Table 3, as the temperature increased from 25 to 55 °C, *Q_m_* estimated by the Langmuir model dropped from 92.59 to 81.30 mg/g, reflecting that lower temperatures favor adsorption. According to the Freundlich model, a rise in adsorption temperature led to a reduction in *K_F_*, reflecting the exothermic nature of the process. The 1/*n* value, ranging from 0.45 to 0.50, indicated that the adsorption occurred easily [35]. Temkin model describes a nearly linear decrease in binding energy between the adsorbate and adsorbent surface as adsorption proceeds. The *K_T_* value decreased with increasing temperature, suggesting that higher temperatures weaken the interaction between the adsorbate and the adsorbent [31]. Moreover, at each temperature, the Langmuir model produced higher *R*^2^ than the Freundlich and Temkin models, indicating that it more accurately described the monolayer adsorption of BOF-TP onto AB-8 resin [35].

**Table 3:** Equations and parameters for three adsorption isotherms.

Models	Equations/Parameters	25°C	35°C	45°C	55°C
Langmuir	Equations	$\frac{C_{e}}{Q_{e}}=0.0108C_{e}+0.7727$	$\frac{C_{e}}{Q_{e}}=0.0109C_{e}+0.9888$	$\frac{C_{e}}{Q_{e}}=0.0118C_{e}+1.6090$	$\frac{C_{e}}{Q_{e}}=0.0123C_{e}+2.2139$
	*K_L_* (L/mg)	0.0139	0.0110	0.0073	0.0056
	*Q_m_* (mg/g)	92.59	91.74	84.75	81.30
	*R*^2^	0.9964	0.9981	0.9919	0.9913

Freundlich	Equations	$\ln Q_{e}=0.4517\ln C_{e}+1.7664$	$\ln Q_{e}=0.4648\ln C_{e}+1.5932$	$\ln Q_{e}=0.4658\ln C_{e}+1.3332$	$\ln Q_{e}=0.4880\ln C_{e}+1.0554$
	*K_F_* [(mg/g)(L/mg)^1/^*^n^*]	5.8498	4.9195	3.7932	2.8731
	1/*n*	0.4517	0.4648	0.4658	0.4880
	*R*^2^	0.9024	0.9252	0.9353	0.9433

Temkin	Equations	$Q_{e}=20.1129\ln C_{e}-39.9561$	$Q_{e}=20.0955\ln C_{e}-45.0818$	$Q_{e}=18.4811\ln C_{e}-48.5137$	$Q_{e}=18.4117\ln C_{e}-54.8609$
	*B_T_* (J/mol)	20.1129	20.0955	18.4811	18.4117
	*K_T_* (L/mg)	0.1372	0.1061	0.0724	0.0508
	*R*^2^	0.9728	0.9838	0.9606	0.9667

### 3.6 Adsorption thermodynamics

As illustrated in Table 4, thermodynamic analysis revealed that the adsorption of BOF-TP onto AB-8 resin was a spontaneous (Δ*G* < 0) and exothermic (Δ*H* < 0) process dominated by physisorption (|Δ*H*| < 43 kJ/mol), as demonstrated by plots of ln(*Q_e_*/*C_e_*) versus *Q_e_* (Fig. 4E) and ln*K_eq_* versus 1/*T* (Fig. 4F) [33]. The diminishing |Δ*G*| with increasing temperature suggested a decline in adsorption spontaneity, whereas the negative Δ*S* indicated increased molecular ordering of phenolics on the AB-8 resin surface [45].

**Table 4:** Adsorption thermodynamic parameters.

Δ*G* (kJ/mol)				Δ*H* (kJ/mol)	Δ*S* (J/mol K)
25°C	35°C	45°C	55°C		
−18.57	−18.56	−17.82	−17.19	–33.09	−48.06

### 3.7 Adsorption kinetics

**Fig. 5:**
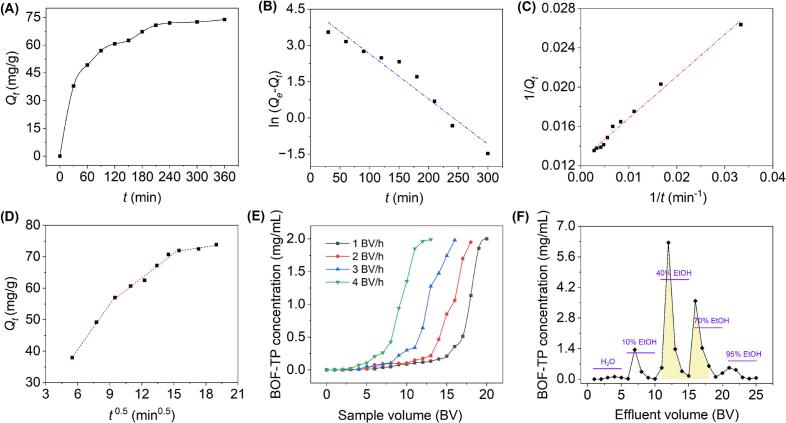
Adsorption kinetics at 25 °C (A), the linear fitting based on the PFO model (B), PSO model (C), and IPD model (D); Breakthrough curves at 1, 2, 3, and 4 BV/h (E) and the gradient elution curve at 2 BV/h (F).

As illustrated in Fig. 5A, *Q_t_* increased sharply during the first 2 h and then gradually slowed, approaching equilibrium at 210 min. This phenomenon was attributed to the initially abundant surface sites that facilitated the binding of phenolic compounds. As the adsorption process continued, factors such as progressive site saturation, increased intermolecular repulsion, and heightened diffusion barriers collectively restricted further adsorption [35].

Kinetic modeling of BOF-TP adsorption on AB-8 resin was performed using the PFO, PSO, and IPD models (Fig. 4B–D). As presented in Table 5, both the PFO and PSO models demonstrated strong agreement with the experimental results, each yielding *R*^2^ values greater than 0.94. Moreover, the theoretical *Q_e_* calculated by the PSO (79.37 mg/g) model was found to be in better agreement with the experimental *Q_e_* (73.84 mg/g) than that calculated by the PFO model (91.09 mg/g). Therefore, in comparison, the PSO model demonstrated a better fit to the experimental data than the PFO model. The adsorption process is typically divided into three sequential steps, diffusion through the boundary layer, migration within the particles, and eventual binding to active sites [31]. The fitting curves obtained from the IPD model displayed multiple stages. None of the plots intersected the origin (*C_i_* > 0), suggesting that the adsorption of phenolics onto AB-8 resin was not governed exclusively by intraparticle diffusion. Instead, boundary layer diffusion or surface adsorption also played a key role in influencing the overall adsorption rate [45].

**Table 5:** Equations and parameters for three adsorption kinetics.

Models	Equations/parameters	
Pseudo-first-order	Equation	$\ln(Q_{e}-Q_{t})=-0.0186t+4.5118$
	*k*_1_	0.0186 min^−1^
	*Q_e_*	91.09 mg/g
	*R*^2^	0.9462

Pseudo-second-order	Equation	$1/Q_{t}=0.4267/t+0.0126$
	*k*_2_	3.7207 × 10^–4^ g/(mg min)
	*Q_e_*	79.37 mg/g
	*R*^2^	0.9888

Intraparticle diffusion (Phase Ⅰ)	Equation	$Q_{t}=4.7767t^{1/2}+11.9174$
	*k_i,_*_1_	4.7767 mg/(g min^1/2^)
	*C_i,_*_1_	11.9174 mg/g
	*R*^2^	0.9991

(Phase Ⅱ)	Equation	$Q_{t}=2.6159t^{1/2}+31.8729$
	*k_i,_*_2_	2.6159 mg/(g min^1/2^)
	*C_i,_*_2_	31.8729 mg/g
	*R*^2^	0.9814

(Phase III	Equation	$Q_{t}=0.5212t^{1/2}+63.7905$
	*k_i,_*_3_	0.5212 mg/(g min^1/2^)
	*C_i,3_*	63.7905 mg/g
	*R*^2^	0.9208

### 3.8 Adsorptive purification

#### 3.8.1 Breakthrough curves

As shown in Fig. 5E, lower flow rates resulted in enhanced adsorption of BOF-TP, which was attributed to the longer contact time between phenolic compounds and the AB-8 resin. In contrast, higher flow rates reduced this interaction period, leading to incomplete adsorption [35]. Similar breakthrough curve patterns were also observed in the purification of phenolics from *Plantago depressa* [33] and *Vernonia patula* (Dryand.) Merr. [45] by NKA-II resin. At flow rates of 1, 2, 3, and 4 BV/h, the sample volumes at breakthrough were 15, 13, 9, and 6 BV, respectively. Considering both throughput and efficiency, 2 BV/h was determined to be optimal for the purification process.

#### 3.8.2 Gradient elution curve

As shown in Fig. 5F, desorption experiments at 2 BV/h showed that both 5 BV of H_2_O and 5 BV of 10 % EtOH were ineffective in eluting phenolics and mainly removed polar impurities. Further chromatographic analysis demonstrated that 40 % EtOH efficiently collected phenolic-rich fractions, while 70 % EtOH partially eluted phenolics. In comparison, 95 % EtOH recovered only small amounts of phenolics because most had already been eluted in previous steps. Based on these results, the optimal collection interval was established at 11 to 18 BV, as indicated by the shaded area in Fig. 5F.

#### 3.8.3 Validation of purification process

Following a systematic investigation, the optimal conditions for purifying BOF-TP using an AB-8 resin column were established as: (1) For adsorption, a 13 BV sample solution containing 2.0 mg/mL BOF-TP was loaded onto the column at 2 BV/h; (2) For desorption, the column was initially washed with H_2_O and 10 % EtOH (5 BV each) at 2 BV/h to remove impurities., followed by sequential elution of phenolic compounds with 5 BV of 30 % ethanol and then 3 BV of 70 % EtOH. After concentrating the collected fractions under vacuum and freeze-drying, a phenolic-rich product was obtained, exhibiting a purity of 75.62 ± 0.95 % and a recovery rate of 82.15 ± 1.63 %. These results confirmed the practicality of the BOF-TP purification protocol based on the chromatography approach established in this study.

### 3.9 UPLC-QTOF-MS/MS analysis

#### 3.9.1 Qualitative analysis

**Fig. 6:**
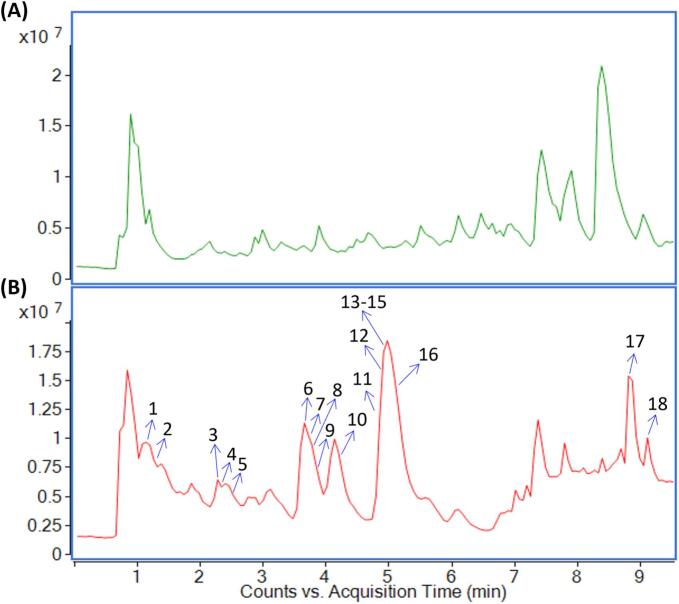
The total ion chromatogram (TIC) of BOF-TP extracts before purification (A) and after purification (B).

The mobile phase, elution protocol, and column temperature were optimized to enhance the performance of UPLC. Fig. 6 shows the total ion chromatogram (TIC) of BOF-TP extracts before and after purification. The results indicated that the purification process successfully eliminated most of the impurities eluting between 7–9 min observed in Fig. 6A. Meanwhile, the chromatographic profile of phenolic compounds were significantly enhanced (Fig. 6B), which validated the effectiveness of the AB-8 macroporous resin for this specific application. A total of 18 phytochemicals were successfully identified by comparison with literature data [48]. Detailed information on the identified compounds, including their retention times, mass errors, and characteristic ions, was summarized in Table 6.

**Table 6:** Compounds identified in the purified BOF-TP extracts using UPLC/Q-TOF-MS/MS.

No.	t_R_ (min)	[M + H]^+^	Formular	Error (ppm)	MS/MS ions (*m*/*z*)	Identification
1	1.202	595.1633	C_27_H_30_O_15_	4.03	403, 421, 439, 457, 523, 577	Apigenin-6,8-di-*C*-glucoside
2	1.261	625.1736	C_28_H_32_O_16_	4.32	409, 439, 469, 487, 511, 535, 571, 607	Diosmetin-6,8-di-*C*-glucoside
3	2.270	597.1795	C_27_H_32_O_15_	3.18	129, 195, 219, 245, 331, 355, 415	Eriocitrin
4	2.289	597.1792	C_27_H_32_O_15_	3.68	195, 219, 245, 355, 417, 485	Neoeriocitrin
5	2.459	595.1637	C_27_H_30_O_15_	3.36	195, 379, 449	Veronicastroside
6	3.640	581.1842	C_27_H_32_O_14_	3.96	129, 147, 153, 435	Naringin
7	3.656	273.0749	C_15_H_12_O_5_	2.93	147, 153, 213, 255	Naringenin
8	3.702	581.1842	C_27_H_32_O_14_	3.96	245, 263, 285, 297, 339, 401	Narirutin
9	3.836	579.1704	C_27_H_30_O_14_	0.69	129, 195, 229, 313, 433	Apigenin-7-*O*-rutinoside
10	4.135	611.1951	C_28_H_34_O_15_	3.11	303, 369, 431, 449	Hesperidin
11	4.854	609.1818	C_28_H_32_O_15_	−0.66	327, 395, 431	Diosmetin-7-*O*-rutinoside
12	4.957	303.0862	C_16_H_14_O_6_	0.33	145, 153, 219, 285	Hesperetin
13	4.963	611.1946	C_28_H_34_O_15_	3.93	303, 315, 345, 395, 449	Neohesperidin
14	4.970	465.1370	C_22_H_24_O_11_	4.51	153, 177, 219, 231, 285, 369	Hesperitin-7-*O*-glucoside
15	4.974	595.2023	C_28_H_34_O_14_	−0.34	153, 219, 287, 353, 379	Neoponcirin
16	5.093	595.2015	C_28_H_34_O_14_	1.01	153, 219, 287, 353, 379	Poncirin
17	8.866	403.1383	C_21_H_22_O_8_	0.99	342, 373	Nobiletin
18	9.106	373.1277	C_20_H_20_O_7_	1.34	297, 325, 358	Tangeretin

#### 3.9.2 Development and validation of the UPLC-QTOF-MS/MS method

As shown in Table 7, linearity, precision, and recovery were within the established acceptable ranges. The method demonstrated strong linearity for all analytes (*R*^2^ > 0.998) throughout the tested concentration range. The LOD and LOQ ranged from 4.86 to 23.97 ng/mL and 11.04 to 73.08 ng/mL, respectively. Both intra- and inter-day precisions (RSD) were less than 3.83 % and 4.96 %. The recovery rates for the seven analytes spanned from 96.86 ± 1.34 % to 102.50 ± 0.97 %. Overall, these results confirm that the developed method is sensitive, accurate, and suitable for the quantitation of seven analytes in BOF.

**Table 7:** Method validation and quantification of seven phenolic compounds in the purified BOF-TP extracts.

Compounds	Regression equation	Linear range (μg/mL)	*R*^2^	LOD (ng/mL)	LOQ (ng/mL)	Precision (RSD, %)		Recovery (%)	Content (%)
						Intra-day	Inter-day		
Eriocitrin	*y* = 5367965*x*-64647	2–10	0.9983	22.02	64.50	1.96	3.05	96.86 ± 1.34	1.27 ± 0.08
Naringin	*y* = 9467842*x* + 104741	4–20	0.9996	4.86	11.04	2.52	3.97	97.74 ± 2.19	6.75 ± 0.12
Naringenin	*y* = 2367462*x*-163754	4–20	0.9992	10.68	35.70	1.86	4.46	99.69 ± 3.15	5.08 ± 0.17
Narirutin	*y* = 1657658*x*-64780	4–10	0.9990	8.93	24.27	2.03	3.65	98.52 ± 3.37	4.13 ± 0.15
Hesperidin	*y* = 53504573*x*-446567	4–20	0.9985	12.21	28.80	2.97	4.96	101.99 ± 2.06	8.11 ± 0.24
Hesperetin	*y* = 8696430*x*-74224	8–40	0.9986	19.06	67.75	3.83	3.04	102.50 ± 0.97	11.26 ± 0.17
Neohesperidin	*y* = 12752997*x* + 952131	8–40	0.9992	23.97	73.08	2.91	4.35	97.35 ± 1.63	18.52 ± 0.10

#### 3.9.3 Quantitative analysis

Seven compounds with good bioactivities, including eriocitrin, naringin, naringenin, narirutin, hesperidin, hesperetin, and neohesperidin, were selected for quantification within the purified BOF-TP extract. As shown in Table 7, neohesperidin constituted the predominant component at 18.52 ± 0.10 %, followed by hesperetin 11.26 ± 0.17 % and hesperidin 8.11 ± 0.24 %. Collectively, these seven compounds accounted for 55.12 % of the total mass of the purified extract. These quantitative results established a reference standard for the quality control of purified BOF-TP extract.

## 4 Conclusions

This study established a novel and environmentally sustainable methodology integrating NADES-UAE with adsorptive purification for the efficient recovery of BOF-TP. ChCl-EG was identified as the optimal solvent. Through BBD optimization, an impressive extraction yield of 104.58 ± 0.34 mg/g was achieved. Among the tested resins, AB-8 exhibited the highest adsorption performance, with the process conforming closely to the Langmuir isotherm and PSO kinetic models. Thermodynamic analysis indicated a spontaneous and exothermic adsorption process accompanied by a reduction in entropy. Subsequent chromatographic purification yielded BOF-TP with a purity of 75.62 ± 0.95 %. Additionally, a UPLC-Q/TOF-MS/MS method was developed for comprehensive chemical profiling and simultaneous quantification of seven major bioactive phenolics, providing a reliable platform for quality assessment. Future studies should focus on scaling up the process to assess its industrial feasibility, particularly regarding energy consumption and economic viability. The health benefits and safety profiles of the enriched extract require validation through *in vitro* and *in vivo* biological assays. Moreover, exploring the application of the purified BOF-TP extract in functional foods or nutraceutical formulations would help translate laboratory-scale success into practical uses.

## CRediT authorship contribution statement

**Ziyu Lv:** Writing – original draft, Investigation, Data curation, Conceptualization. **Jing Ma:** Writing – original draft, Methodology, Investigation, Funding acquisition, Data curation. **Chi Wei:** Writing – original draft, Methodology, Investigation, Data curation. **Jiaqi Wang:** Project administration, Formal analysis. **Dan Wang:** Formal analysis, Conceptualization. **Xinxin Cheng:** Validation, Supervision. **Guoliang Chen:** Project administration. **Luis A.J. Mur:** Formal analysis, Conceptualization. **Yanfeng Wang:** Writing – review & editing, Supervision. **Duo Cao:** Writing – review & editing, Supervision, Resources, Funding acquisition.

## Declaration of competing interest

The authors declare that they have no known competing financial interests or personal relationships that could have appeared to influence the work reported in this paper.

## References

[1] Rownaghi M., Niakousari M. (2024). Sour orange (*Citrus aurantium*) seed, a rich source of protein isolate and hydrolysate–a thorough investigation. Heliyon.

[2] Ren W., Wang S., Zhang J., Liu D. (2024). Ethnopharmacology, chemical composition and functions of *Citrus aurantium* L. J. Food Meas. Charact..

[3] Aboualsoltani F., Bastani P., Khodaie L., Fazljou S.M.B., Salehi-Pourmehr H. (2024). The effect of *Citrus aurantium* L. flower extract on the severity of primary dysmenorrhoea: a double-blind, randomised, controlled clinical trial. J. Herb. Med..

[4] Rahnama S., Rabiei Z., Alibabaei Z., Mokhtari S., Rafieian-Kopaei M., Deris F. (2015). Anti-amnesic activity of *Citrus aurantium* flowers extract against scopolamine-induced memory impairments in rats. Neurol. Sci..

[5] Değirmenci H., Erkurt H. (2020). Relationship between volatile components, antimicrobial and antioxidant properties of the essential oil, hydrosol and extracts of *Citrus aurantium* L. flowers. J. Infec. Public Heal..

[6] Değirmenci H., Erkurt H. (2020). Chemical profile and antioxidant potency of *Citrus aurantium* L. flower extracts with antibacterial effect against foodborne pathogens in rice pudding. LWT - Food Sci. Technol..

[7] Khodabakhsh P., Shafaroodi H., Asgarpanah J. (2015). Analgesic and anti-inflammatory activities of *Citrus aurantium* L. blossoms essential oil (neroli): involvement of the nitric oxide/cyclic-guanosine monophosphate pathway. J. Nat. Med..

[8] Kang S.R., Park K.I., Park H.S., Lee D.H., Kim J.A., Nagappan A., Kim E.H., Lee W.S., Shin S.C., Park M.K., Han D.Y., Kim G.S. (2011). Anti-inflammatory effect of flavonoids isolated from Korea *Citrus aurantium* L. on lipopolysaccharide-induced mouse macrophage RAW 264.7 cells by blocking of nuclear factor-kappa B (NF-κB) and mitogen-activated protein kinase (MAPK) signalling pathways. Food Chem..

[9] Karimi E., Oskoueian E., Hendra R., Oskoueian A., Jaafar H.Z. (2012). Phenolic compounds characterization and biological activities of *Citrus aurantium* bloom. Molecules.

[10] Cai W.F., Yan M.M., Wang Z., Jiang M.P., Yan B., Shen C.Y. (2022). Optimization of the extract from flower of *Citrus aurantium* L. var. amara Engl. and its inhibition of lipid accumulation. J. Food Biochem..

[11] Hsouna A.B., Hamdi N., Halima N.B., Abdelkafi S. (2013). Characterization of essential oil from *Citrus aurantium* L. flowers: antimicrobial and antioxidant activities. J. Oleo Sci..

[12] Shen C.Y., Yang L., Jiang J.G., Zheng C.Y., Zhu W. (2017). Immune enhancement effects and extraction optimization of polysaccharides from *Citrus aurantium* L. var. amara Engl. Food Funct..

[13] Qi X., Liu H., Ren Y., Zhu Y., Wang Q., Zhang Y., Wu Y., Yuan L., Yan H., Liu M. (2023). Effects of combined binding of chlorogenic acid/caffeic acid and gallic acid to trypsin on their synergistic antioxidant activity, enzyme activity and stability. Food Chem.: X.

[14] Addi M., Elbouzidi A., Abid M., Tungmunnithum D., Elamrani A., Hano C. (2021). An overview of bioactive flavonoids from *Citrus* fruits. Appl. Sci..

[15] Chaudhary K., Khalid S., Zahid M., Ansar S., Zaffar M., Hassan S.A., Naeem M., Maan A.A., Aadil R.M. (2024). Emerging ways to extract lycopene from waste of tomato and other fruits, a comprehensive review. J. Food Process Eng..

[16] Khalid S., Chaudhary K., Amin S., Raana S., Zahid M., Naeem M., Khaneghah A.M., Aadil R.M. (2024). Recent advances in the implementation of ultrasound technology for the extraction of essential oils from terrestrial plant materials: a comprehensive review. Ultrason. Sonochem..

[17] Khalid S., Chaudhary K., Aziz H., Amin S., Sipra H.M., Ansar S., Rasheed H., Naeem M., Onyeaka H. (2025). Trends in extracting protein from microalgae *Spirulina platensis*, using innovative extraction techniques: mechanisms, potentials, and limitations. Crit. Rev. Food Sci..

[18] Chaudhary K., Khalid S., AlMasoud N., Alomar T.S., Ansar S., Ghazal A.F., Aït-Kaddour A., Aadil R.M. (2025). Impact of ultrasonication, ozonation, and their combination on the preservation of novel clean-label functional drink of strawberry-cantaloupe incorporated with *Spirulina platensis* and orange peel extracts. Ultrason. Sonochem..

[19] Khadhraoui B., Ummat V., Tiwari B.K., Fabiano-Tixier A.S., Chemat F. (2021). Review of ultrasound combinations with hybrid and innovative techniques for extraction and processing of food and natural products. Ultrason. Sonochem..

[20] Hou M., Lin C., Zhu L., Bian Z. (2025). Phenolics from *Chaenomeles speciosa* leaves: Ionic liquid-based ultrasound-assisted extraction, adsorptive purification, UPLC–QqQ–MS/MS quantification, and bioactivity assessment. Ultrason. Sonochem..

[21] Perna F.M., Vitale P., Capriati V. (2020). Deep eutectic solvents and their applications as green solvents. Curr. Opin. Green Sust..

[22] Farooq M.Q., Abbasi N.M., Smith E.A., Petrich J.W., Anderson J.L. (2022). Characterizing the solvation characteristics of deep eutectic solvents composed of active pharmaceutical ingredients as a hydrogen bond donor and/or acceptor. ACS Sustain. Chem. Eng..

[23] Prabhune A., Dey R. (2023). Green and sustainable solvents of the future: deep eutectic solvents. J. Mol. Liq..

[24] Liu Y., Zhe W., Zhang R., Peng Z., Wang Y., Gao H., Guo Z., Xiao J. (2022). Ultrasonic-assisted extraction of polyphenolic compounds from *Paederia scandens* (Lour.) Merr. using deep eutectic solvent: optimization, identification, and comparison with traditional methods. Ultrason. Sonochem..

[25] Kaur K., Schmitt-Kopplin P., Malik A.K. (2024). Green and efficient extraction of phenolic compounds from Neem leaves using deep eutectic solvents based ultrasonic-assisted extraction. Food Chem..

[26] Chaudhary K., Khalid S., Alomar T.S., AlMasoud N., Ansar S., Ghazal A.F., Aït-Kaddour A., Aadil R.M. (2025). Ultrasound assisted natural deep eutectic solvents based sustainable extraction of *Spirulina platensis* and orange peel extracts for the development of strawberry-cantaloupe based novel clean-label functional drink. Ultrason. Sonochem..

[27] Rashid R., Wani S.M., Manzoor S., Masoodi F.A., Dar M.M. (2023). Green extraction of bioactive compounds from apple pomace by ultrasound assisted natural deep eutectic solvent extraction: optimisation, comparison and bioactivity. Food Chem..

[28] Aljawarneh R.Y.A., Che Zain M.S., Zakaria F. (2024). Macroporous polymeric resin for the purification of flavonoids from medicinal plants: a review. J. Sep. Sci..

[29] Fu L., Zuo J., Liao K., Shao M., Si W., Zhang H., Gu F., Huang W., Li B., Shao Y. (2023). Preparation of adsorption resin and itas application in VOCs adsorption. J. Polym. Res..

[30] Shi T., Ma L., Xi X., Nie Z. (2024). Preparation of functional polytertiary amine macroporous resin and its adsorption and separation properties for tungsten and molybdenum. Sep. Purif. Technol..

[31] Hou M., Zhou Y., Lin C., Shi J., Hou H., Ma Y., Zhu L., Bian Z. (2025). Green valorization of Chaenomelis Fructus agro-industrial by-products as a source of phenolics: ultrasound-assisted extraction, adsorptive enrichment and quality control. Food Chem..

[32] Yang M., Li J., Zhao C., Xiao H., Fang X., Zheng J. (2023). LC-Q-TOF-MS/MS detection of food flavonoids: Principle, methodology, and applications. Crit. Rev. Food Sci..

[33] Cao D., Bu F., Cheng X., Zhao C., Yin Y., Liu P. (2024). Purification of phenolics from *Plantago depressa* by macroporous resin: Adsorption/desorption characteristics, chromatographic process development and UPLC-TQ-MS/MS quantitative analysis. LWT – Food Sci. Technol..

[34] Patil S.S., Pathak A., Rathod V.K. (2021). Optimization and kinetic study of ultrasound assisted deep eutectic solvent based extraction: a greener route for extraction of curcuminoids from *Curcuma longa*. Ultrason. Sonochem..

[35] M. Hou, W. Hu, Z. Xiu, Y. Shi, K. Hao, D. Cao, Y. Guan, H. Yin, 2020. Efficient enrichment of total flavonoids from *Pteris ensiformis* Burm. extracts by macroporous adsorption resins and *in vitro* evaluation of antioxidant and antiproliferative activities. J. Chromatogr. B 1138, 121960. doi: 10.1016/j.jchromb.2019.121960. 10.1016/j.jchromb.2019.121960 31918307

[36] Wei H., Wang X., Wang J., Ren S., Mur L.A., Lu D., Cao D. (2025). Flavonoids from sour jujube leaves: Ultrasound-assisted extraction, UPLC-QQQ-MS/MS quantification, and ameliorative effect on DSS-induced ulcerative colitis in mice. Ultrason. Sonochem..

[37] Zhou P., Wang X., Liu P., Huang J., Wang C., Pan M., Kuang Z. (2018). Enhanced phenolic compounds extraction from *Morus alba* L. leaves by deep eutectic solvents combined with ultrasonic-assisted extraction. Ind. Crop Prod..

[38] Serna-Vázquez J., Ahmad M.Z., Boczkaj G., Castro-Muñoz R. (2021). Latest insights on novel deep eutectic solvents (DES) for sustainable extraction of phenolic compounds from natural sources. Molecules.

[39] Zheng B., Yuan Y., Xiang J., Jin W., Johnson J.B., Li Z., Wang C., Luo D. (2022). Green extraction of phenolic compounds from foxtail millet bran by ultrasonic-assisted deep eutectic solvent extraction: Optimization, comparison and bioactivities. LWT – Food Sci. Technol..

[40] Deniz S., Ünlü A.E., Takaç S. (2023). Ultrasound-assisted natural deep eutectic solvent extraction of phenolic compounds from apple pomace. Sep. Sci. Technol..

[41] Cao Y., Song Z., Dong C., Ni W., Xin K., Yu Q., Han L. (2023). Green ultrasound-assisted natural deep eutectic solvent extraction of phenolic compounds from waste broccoli leaves: optimization, identification, biological activity, and structural characterization. LWT – Food Sci. Technol..

[42] Wang S., Feng Y., Yu X., Yang Z., Jiao P., Niu Q. (2025). Integrated deep eutectic solvent extraction and resin adsorption for recovering polyphenols from *citrus reticulata* Blanco peels: Process optimization, compositional analysis, and activity determination. Sep. Purif. Technol..

[43] Liu D., Liu H., Fu X., Guo S., Fan S., Fang X. (2025). Effective extraction and separation of bioactive lignans from *Forsythia suspensa* leaves using deep eutectic solvents coupled with macroporous resin. Microchem. J..

[44] Hou M., Shi J., Lin C., Zhu L., Bian Z. (2024). Ultrasound-assisted extraction of triterpenoids from *Chaenomeles speciosa* leaves: process optimization, adsorptive enrichment, chemical profiling, and protection against ulcerative colitis. Ultrason. Sonochem..

[45] Hou M., Zhang L. (2021). Adsorption/desorption characteristics and chromatographic purification of polyphenols from *Vernonia patula* (Dryand.) Merr. using macroporous adsorption resin. Ind. Crop Prod..

[46] Lu Y., Liu Y., Chang X., Bai L., Bai Y., Qi H. (2025). Efficient purification of polyphenols from *Ascophyllum nodosum* using macroporous resin: adsorption mechanism, kinetics, and enhanced bioactivity. Sep. Purif. Technol..

[47] Guo L., Qiang T., Ma Y., Ren L., Dai T. (2021). Purification and characterization of hydrolysable tannins extracted from *Coriaria nepalensis* bark using macroporous resin and their application in gallic acid production. Ind. Crop Prod..

[48] Gao L., Gou N., Amakye W.K., Wu J., Ren J. (2022). Bioactivity guided isolation and identification of phenolic compounds from *Citrus aurantium* L. with anti-colorectal cancer cells activity by UHPLC-Q-TOF/MS. Curr. Res. Food Sci..

