# Supplementary material for: Green recovery of phenolics from bitter orange flowers: Natural deep eutectic solvent-ultrasound synergistic extraction, adsorptive purification, and UPLC/Q-TOF-MS/MS analysis
Source: Ultrason Sonochem. 2025 Oct 3;122:107597. doi: 10.1016/j.ultsonch.2025.107597 (PMC12528869; doi:10.1016/j.ultsonch.2025.107597)
Supplement: Supplementary Data 1 [file mmc1.docx]

Supplementary Material for

**Green recovery of phenolics from bitter orange flowers: Natural deep eutectic solvent-ultrasound synergistic extraction, adsorptive purification, and UPLC/Q-TOF-MS/MS analysis**

Ziyu Lv ^a, 1^, Jing Ma ^b, 1^ Chi Wei ^c, 1^, Jiaqi Wang ^a^, Dan Wang ^a^, Xinxin Cheng ^a^, Guoliang Chen ^a^, Luis A. J. Mur ^d^, Yanfeng Wang ^a,^*, Duo Cao ^a,^*

*^a^ Shaanxi Key Laboratory of Research and Utilization of Resource Plants on the Loess Plateau, College of Life Sciences, Yan’an University, Yan’an 716000, Shaanxi, China*

*^b^ Department of Pathology, Xijing Hospital and School of Basic Medicine, Fourth Military Medical University, Xi’an, China*

*^c^ Department of Cardiovascular Medicine, No.986 Hospital, Xijing Hospital, Air Force Military Medical University, Xi’an, China*

*^d^ Department of Life Sciences, Aberystwyth University, Ceredigion SY23 3DA, UK*

*^1^ These authors contributed equally to this work.*

** Corresponding authors:*

*E-mail address: [waf@yau.edu.cn](mailto:waf@yau.edu.cn) (Y. Wang); [duocao@yau.edu.cn](mailto:duocao@yau.edu.cn) (D. Cao);*

**Table S1**

Specifications for the seven resins tested.

| Resins | Particle size  (mm) | Polarity | Specific surface area  (m^2^/g) | Average pore diameter  (nm) |
| --- | --- | --- | --- | --- |
| D101 | Non-polar | 0.3–1.25 | 480-520 | 25-28 |
| HPD-100 | Non-polar | 0.3–1.25 | 650-700 | 9-10 |
| AB-8 | Weak-polar | 0.3–1.25 | 480-520 | 13-14 |
| HPD-400A | Medium-polar | 0.3~1.20 | 500~550 | 8.5-9.0 |
| S-8 | Medium-polar | 0.3–1.20 | 500-600 | 40-45 |
| DM301 | Polar | 0.3–1.25 | 500-550 | 10-12 |
| DA201 | Strong-polar | 0.3~1.25 | 500~550 | 10~12 |

**Table S2**

Single-factor experimental design.

| Variables | Levels | Fixed conditions |
| --- | --- | --- |
| Water content (%) | 20, 30, 40, 50, 60, 70 | 30 min, 65℃, 25 mL/g, 300 W |
| Liquid-to-solid ratio (mL/g) | 10, 15, 20, 25, 30 | 50%, 30 min, 65℃, 300 W |
| Ultrasonic power (W) | 200, 250, 300, 350, 400, 450 | 50%, 30 min, 65℃, 25 mL/g |
| Extraction time (min) | 10, 20, 30, 40, 50, 60 | 50%, 65℃, 25 mL/g, 300 W |
| Extraction temperature (℃) | 25, 35, 45, 55, 65, 75 | 50%, 30 min, 25 mL/g, 300 W |

**Table S3**

The actual and coded levels of the independent variables used for BBD.

| Independent variables | Coded symbols | Levels | | |
| --- | --- | --- | --- | --- |
|  |  | -1 | 0 | 1 |
| Water content (%) | *X*_1_ | 30 | 40 | 50 |
| Liquid-to-solid ratio (mL/g) | *X*_2_ | 15 | 20 | 25 |
| Ultrasonic power (W) | *X*_3_ | 300 | 350 | 400 |
| Extraction time (min) | *X*_4_ | 30 | 40 | 50 |

**Table S4**

Box-Behnken design and results.

| Run | *X*_1_ (%) | *X*_2_ (mL/g) | *X*_3_ (W) | *X*_4_ (min) | *Y* (mg/g) |
| --- | --- | --- | --- | --- | --- |
| 1 | 40 | 20 | 400 | 30 | 87.16 |
| 2 | 50 | 20 | 400 | 40 | 80.89 |
| 3 | 40 | 15 | 350 | 30 | 96.08 |
| 4 | 40 | 20 | 350 | 40 | 102.65 |
| 5 | 50 | 25 | 350 | 40 | 81.54 |
| 6 | 40 | 15 | 350 | 50 | 101.43 |
| 7 | 40 | 20 | 350 | 40 | 104.03 |
| 8 | 30 | 20 | 350 | 30 | 90.55 |
| 9 | 40 | 20 | 300 | 50 | 93.42 |
| 10 | 50 | 15 | 350 | 40 | 87.24 |
| 11 | 40 | 15 | 300 | 40 | 99.53 |
| 12 | 30 | 15 | 350 | 40 | 96.07 |
| 13 | 40 | 20 | 300 | 30 | 91.52 |
| 14 | 40 | 25 | 300 | 40 | 87.03 |
| 15 | 40 | 20 | 350 | 40 | 102.98 |
| 16 | 50 | 20 | 350 | 50 | 87.29 |
| 17 | 30 | 25 | 350 | 40 | 86.58 |
| 18 | 40 | 20 | 400 | 50 | 93.55 |
| 19 | 50 | 20 | 300 | 40 | 85.35 |
| 20 | 50 | 20 | 350 | 30 | 85.10 |
| 21 | 40 | 15 | 400 | 40 | 92.14 |
| 22 | 30 | 20 | 350 | 50 | 97.01 |
| 23 | 40 | 20 | 350 | 40 | 102.39 |
| 24 | 40 | 25 | 350 | 50 | 91.07 |
| 25 | 30 | 20 | 400 | 40 | 92.57 |
| 26 | 40 | 25 | 350 | 30 | 88.07 |
| 27 | 40 | 20 | 350 | 40 | 102.69 |
| 28 | 30 | 20 | 300 | 40 | 88.97 |
| 29 | 40 | 25 | 400 | 40 | 90.29 |

*Notes*: *X*_1_ – Water content; *X*_2_ – liquid-to-solid ratio; *X*_3_ – ultrasonic power; *X*_4_ – extraction time; *Y* – BOF-TP yield

**Figure S1**


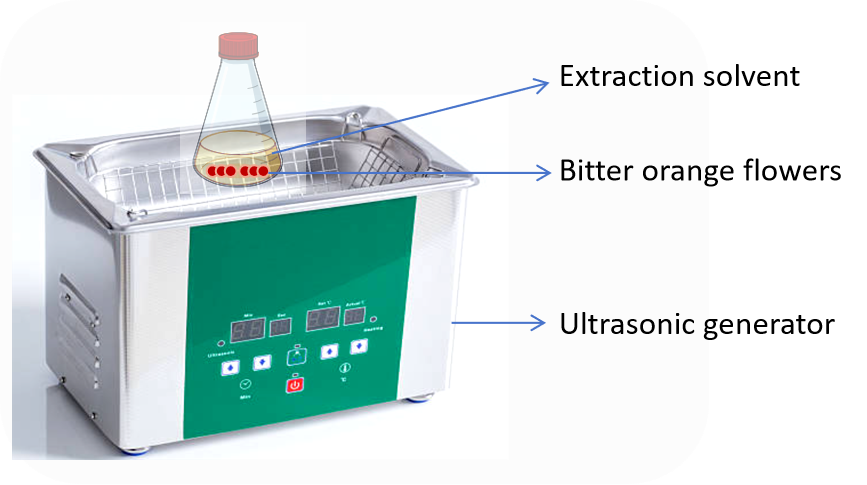


**Fig. S1** Schematic of the extraction process.

- **Pre-treatment of resins**:

Newly purchased resins were immersed in ethanol for 12 h and then washed with water. They were subsequently soaked in a 1.3 mol/L HCl solution for another 12 h, followed by thorough washing with water. Next, the resins were submerged in a 4 g/100 mL NaOH solution for 12 h and again washed thoroughly with water. Finally, the resins were dried at 80°C until they reached a constant weight. Before use, the dried resins were reactivated by soaking them in EtOH overnight and rinsing thoroughly with water.

- **A quadratic polynomial model was derived from the BBD results using multiple regression analysis and was expressed in coded factors as follows**:

$\begin{matrix} Y=102.95-3.70X_{1}-3.99X_{2}-0.7683X_{3}+2.11X_{4}+0.9475X_{1}X_{2}-2.01X_{1}X_{3} \\ \text{ }-1.07X_{1}X_{4}+2.66X_{2}X_{3}-0.5875X_{2}X_{4}+1.12X_{3}X_{4}-9.51X_{1}^{2}-4.78X_{2}^{2} \\ \text{ }-6.61X_{3}^{2}-4.13X_{4}^{2} \end{matrix}$ (12)

- **Determination of total phenolic content**

Specifically, 50 μL of sample extract was reacted with 200 μL of 10-fold diluted Folin-Ciocalteu reagent. After incubation for 3 min, 1 mL of 7.5% (w/v) Na_2_CO_3_ solution was added. The resulting mixture was vortexed and then incubated for 1 h at room temperature in the dark. The absorbance (*A*) at 760 nm was recorded, and total phenolic concentration (*C*) was measured against a gallic acid calibration curve (*A* = 0.0022*C* + 0.0635, *R*² = 0.9997) over a range of 20–400 μg/mL.
